# Overexpression of P70 S6 kinase protein is associated with increased risk of locoregional recurrence in node-negative premenopausal early breast cancer patients

**DOI:** 10.1038/sj.bjc.6601741

**Published:** 2004-03-30

**Authors:** J A van der Hage, L J C M van den Broek, C Legrand, P C Clahsen, C J A Bosch, E C Robanus-Maandag, C J H van de Velde, M J van de Vijver

**Affiliations:** 1 Department of Surgery, Leiden University Medical Center, Albinusdreef 2, 2300 RC Leiden, The Netherlands; 2 Department of Pathology, Leiden University Medical Center, Albinusdreef 2, 2300 RC Leiden, The Netherlands; 3 EORTC Data Center, Avenue E. Mounier 83 b11, 1200 Brussels, Belgium; 4 Department of Pathology and Division of Experimental Therapy, Netherlands Cancer Institute, Plesmanlaan 121, 1066 CX Amsterdam, The Netherlands

**Keywords:** node-negative, premenopausal, early-stage breast cancer, P70 S6 kinase protein, locoregional recurrence

## Abstract

The RPS6KB1 gene is amplified and overexpressed in approximately 10% of breast carcinomas and has been found associated with poor prognosis. We studied the prognostic significance of P70 S6 kinase protein (PS6K) overexpression in a series of 452 node-negative premenopausal early-stage breast cancer patients (median follow-up: 10.8 years). Immunohistochemistry was used to assess PS6K expression in the primary tumour, which had previously been analysed for a panel of established prognostic factors in breast cancer. In a univariate analysis, PS6K overexpression was associated with worse distant disease-free survival as well as impaired locoregional control (HR 1.80, *P* 0.025 and HR 2.50, *P* 0.006, respectively). In a multivariate analysis including other prognostic factors, PS6K overexpression remained an independent predictor for poor locoregional control (RR 2.67, *P* 0.003). To our knowledge, P70 S6 kinase protein is the first oncogenic marker that has prognostic impact on locoregional control and therefore may have clinical implications in determining the local treatment strategy in early-stage breast cancer patients.

The treatment of breast cancer is guided by risk factors. Approximately 70% of all node-negative breast cancer patients can be cured by locoregional therapy alone. This automatically implies that the remaining 30% of these patients will develop a recurrence despite adequate locoregional therapy. Currently, treatment strategy in breast cancer is based upon tumour stage, grade, and hormone receptor status. Additional prognostic factors are greatly needed, first to select those patients who might benefit from adjuvant systemic therapy and second to optimise locoregional therapy in order to avoid locoregional recurrences.

The prognostic significance of a considerable number of tumour markers has already been investigated but to date, none of these factors can be used to guide the treatment of primary breast cancer.

A recent study by Barlund *et al* (2000a) demonstrated that amplification of a putative tumour marker called P70 S6 kinase protein (PS6K) might be associated with poor outcome in breast cancer. In addition, the authors reported that RPS6KB1 gene amplification and PS6K overexpression are significantly correlated.

The RPS6KB1 gene is located at 17q23 and amplified in approximately 10% of all primary breast cancer cases. PS6K is a ribosomal protein that is involved in the progression from the G1 to S phase of the cell cycle. It is rapidly activated in response to mitogenic stimuli, for example, growth factors, cytokines, and oncogene products (Grove *et al*, 1991; Lane *et al*, 1993; Chou *et al*, 1995; Grammer *et al*, 1996; Thomas *et al*, 1997; Couch *et al*, 1999; Barlund *et al*, 2000b; Wu *et al*, 2000; Latham *et al*, 2001; Monni *et al*, 2001; Andersen *et al*, 2002; Li *et al*, 2002; Sinclair *et al*, 2002; Sinclair *et al*, 2003).

To study the significance of P70 S6 kinase protein compared with other established prognostic factors in breast cancer, we have tested the prognostic significance of PS6K overexpression in a subset of node-negative premenopausal early breast cancer patients. In this series, we have shown previously that premenopausal node-negative breast cancer patients whose tumours show p53 accumulation have a poor response to one cycle of adjuvant chemotherapy, whereas patients whose tumours have no accumulation of p53 benefit from adjuvant chemotherapy. In addition, we showed Ki-67 overexpression, negative ER status, and young age (<43 years) to be associated with worse prognosis (Clahsen *et al*, 1998).

## PATIENTS AND METHODS

### Patients

All patients were drawn from EORTC trial 10854 (1986–1991, median follow up 10.8 years). This trial, which randomised 2795 patients, studied whether one course of peri-operative chemotherapy given directly after surgery yields better results in terms of treatment outcome than surgery alone. Peri-operative chemotherapy consisted of one single course of doxorubicin 50 mg m^−2^, 5-fluorouracil 600 mg m^−2^, and cyclophosphamide 600 mg m^−2^ (FAC), administered intravenously within 36 h after surgery. Axillary lymph node-positive premenopausal patients in the peri-operative chemotherapy group were recommended to receive five additional cycles of cyclophosphamide, methotrexate, and 5-fluorouracil (CMF) postoperatively. Node-positive patients, younger than 50 years, who did not receive peri-operative chemotherapy, were advised to be treated with one conventional course of FAC followed by five cycles of CMF after surgery. At randomisation, patients were stratified for institution, age (⩽50 years or >50 years), and surgical procedure (breast conserving therapy and modified radical mastectomy). Prolonged adjuvant systemic treatment was left to the discretion of the local investigator (van der Hage *et al*, 2001).

In total, 676 node-negative premenopausal patients were enrolled in this trial and representative tumour material was collected for 452 patients. Tumour material consisted of formalin-fixed, paraffin-embedded tumour blocks. Tumours were histologically typed and graded (Elston *et al*, 1991) centrally by one pathologist; immunohistochemistry to assess the expression of various proteins has been performed. Results of these studies have been reported previously (Clahsen *et al*, 1998). For the present study, assays were reviewed simultaneously by two investigators (MJ Van de Vijver, JA Van der Hage) who had to come to an agreement in case of any uncertainties. During the evaluation of the results, the investigators were blinded for the clinical outcome of the patients.

### P70 S6 kinase protein expression

A standard indirect immunoperoxidase protocol with a 3,3′-diaminobenzidine/imidazole solution as a chromogen was used. Before incubation with the primary antibody, antigen retrieval was done by boiling the sections in 10 mM citrate buffer for 10 min using a microwave oven. PS6K expression was determined using a polyclonal anti-p70 s6k antibody (sc-230 Santa Cruz Biotechnology, Santa Cruz, USA) (1 : 1000 dilution in PBS containing 1% bovine serum albumin). PS6K staining was evaluated in tumour cells and in normal ductal epithelial cells. PS6K staining was scored categorical as: 0=no staining; 1=weak cytoplasmic staining; 2=moderate cytoplasmic staining; 3=strong cytoplasmic staining. In all cases analysed, the staining was homogeneously distributed in the normal cells and also in the tumour cells. If the difference in staining score between the tumour cells and the normal epithelial ducts was greater or equal than two, tumours were deemed PS6K positive.

### RPS6KB1 gene amplification

Two-colour FISH of tumor interphase nuclei was performed according to the ERBB2 short protocol of Ventana Medical Systems, Inc. (Tucson, AZ, USA). The Spectrum Orange-labelled chromosome 17 centromeric probe was purchased from Vysis, Inc. (Downers Grove, IL, USA), the unlabelled bacterial artificial chromosome (BAC) clones for PS6K was isolated from a BAC library (RPCI-13 BAC library, Research Genetics, Inc.). Fluorescent signals were counted in 2 × 20 non-overlapping nuclei per component. Mapping of the PS6K BAC was verified by FISH on metaphase chromosomes.

### Other tumour markers

Previously, tumour sections had been stained and analysed for oestrogen and progesterone receptor status, Ki-67 positivity, P53 expression, HER2 expression, and mitotic index (Clahsen *et al*, 1998).

### Statistical methods

This analysis was based on locoregional control, distant-disease free survival, and overall survival. Locoregional recurrence was defined as the time to locoregional recurrence as a first event. Locoregional recurrences occurring simultaneously or after the diagnosis of distant metastasis or contralateral breast cancer or a secondary primary tumour were censored. Distant disease-free survival was defined as the time to distant metastasis or death, whichever of the events happened first. All variables were first analysed for their prognostic importance in a univariate analysis.

Eight potential prognostic variables were considered: PS6K (negative *vs* positive), ER status (negative *vs* positive), PgR status (negative *vs* positive), HER2 overexpression (negative *vs* positive), Ki67 (negative, i.e. ⩽20% of positive tumour cells, *vs* positive, >20% positive tumour cells), histologic tumour grade (grade I *vs* grade II *vs* grade III), tumour diameter (*T*⩽2 cm *vs T*>2 cm), and p53 (negative *vs* positive).

To test the independent prognostic significance of PS6K overexpression, we included PS6K together with the previously tested markers into a multivariate Cox regression analysis for overall survival, progression-free survival, distant disease-free survival, and locoregional control. Only markers that were significant predictors in the univariate analysis were included in the multivariate analysis.

A Cox proportional hazards model was used for the univariate and multivariate analyses (Cox, 1972). For factors with only two levels the second one was compared to the first one, while for factors with more than two levels dummy variables were used to compare each level to the first one. Patients who had missing information for any of the variables in the analysis were excluded when this variable was included in the model. All tests were two-sided with a 5% alpha level.

## RESULTS

Patient characteristics are listed in Table 1

**Table 1:** Patient characteristics

	***N*=452**
*Age (year)*	
Median (range)	44 (24–63)
	
*Local treatment* (*N* (%))	
Breast-conserving therapy	368 (81)
Mastectomy	84 (19)
	
*Tumour diameter* (*N* (%))	
⩽2 cm	278 (62)
>2 cm	148 (33)
Unknown	26 (6)
	
*Histologic tumour type* (*N* (%))	
Infiltrating ductal	316 (70)
Infiltrating lobular	34 (8)
Other	91 (20)
Unknown	11 (2)
	
*Histologic tumour grade* (*N* (%))	
I	155 (34)
II	144 (32)
III	131 (21)
Unknown	22 (5)
	
*ER status* (*N* (%))	
Positive	390 (86)
Negative	46 (10)
Unknown	16 (4)
	
*PgR status* (*N* (%))	
Positive	329 (73)
Negative	106 (23)
Unknown	17 (4)
	
*HER2 overexpression* (*N* (%))	
Negative	380 (84)
Positive	60 (13)
Unknown	12 (3)
	
*p53 expression* (*N* (%))	
Negative	359 (79)
Positive	81 (18)
Unknown	12 (3)
	
*Ki-67* (*N* (%))	
Negative	217 (48)
Positive	215 (48)
Unknown	20 (4)
	
*PS6K*	
Negative	391 (87)
Positive	39 (9)
Unknown	22 (5)

. At the time of the analysis, the median follow-up period was 10.8 years, 80 (18%) of the 452 patients had died, 126 (29%) patients had experienced distant metastases or death, and 67 (15%) patients experienced a locoregional recurrence as first event (see Table 2

**Table 2:** Event rates

		**Number of events**		
**Patient characteristics**	***N*=452**	**Overall survival**	**Locoregional recurrence (first event)**	**Distant disease-free survival**
*Local treatment* (*N* (%))				
Breast-conserving therapy	368 (81)	65 (18)	58 (16)	102 (28)
Mastectomy	84 (19)	15 (18)	9 (11)	24 (29)
				
*Tumour diameter* (*N* (%))				
⩽2 cm	278 (62)	34 (12)	41 (15)	68 (24)
>2 cm	148 (33)	40 (27)	21 (14)	51 (34)
Unknown	26 (6)	6 (23)	5 (19)	7 (27)
				
*Histologic tumour type* (*N* (%))				
Infiltrating ductal	316 (70)	60 (19)	48 (15)	91 (29)
Infiltrating lobular	34 (8)	4 (12)	9 (26)	7 (21)
Other	91 (20)	14 (15)	8 (9)	25 (27)
Unknown	11 (2)	2 (18)	2 (18)	3 (27)
				
*Histologic tumour grade* (*N* (%))				
I	155 (34)	10 (6)	23 (15)	30 (19)
II	144 (32)	30 (21)	27 (19)	45 (31)
III	131 (21)	36 (27)	14 (11)	47 (36)
Unknown	22 (5)	4 (18)	3 (14)	4 (18)
				
*ER status* (*N* (%))				
Positive	390 (86)	64 (16)	60 (15)	104 (27)
Negative	46 (10)	13 (28)	4 (9)	18 (39)
Unknown	16 (4)	3 (19)	3 (19)	4 (25)
				
*PgR status* (*N* (%))				
Positive	329 (73)	5 (16)	53 (16)	90 (27)
Negative	106 (23)	27 (25)	12 (11)	33 (31)
Unknown	17 (4)	2 (12)	2 (12)	3 (18)
				
*HER2 overexpression* (*N* (%))				
Negative	380 (84)	66 (17)	52 (14)	107 (28)
Positive	60 (13)	12 (20)	13 (22)	16 (27)
Unknown	12 (3)	2 (17)	2 (17)	3 (25)
				
*P53 expression* (*N* (%))				
Negative	359 (79)	59 (16)	47 (13)	99 (28)
Positive	81 (18)	19 (23)	18 (22)	24 (30)
Unknown	12 (3)	2 (17)	2 (17)	3 (25)
				
Ki-67 (N (%))				
Negative	217 (48)	19 (9)	36 (17)	43 (20)
Positive	215 (48)	59 (27)	27 (13)	78 (36)
Unknown	20 (4)	2 (10)	4 (20)	5 (25)
				
*PS6K*				
Negative	391 (87)	66 (17)	52 (13)	102 (26)
Positive	39 (9)	8 (21)	11 (28)	17 (44)
Unknown	22 (5)	6 (27)	4 (18)	7 (32)

). PS6K expression levels could be assessed in 430 tumours. In all, 39 tumours (9%) showed PS6K overexpression (Table 1). Examples of PS6K overexpression are shown in Figure 1A & B

**Figure 1:**
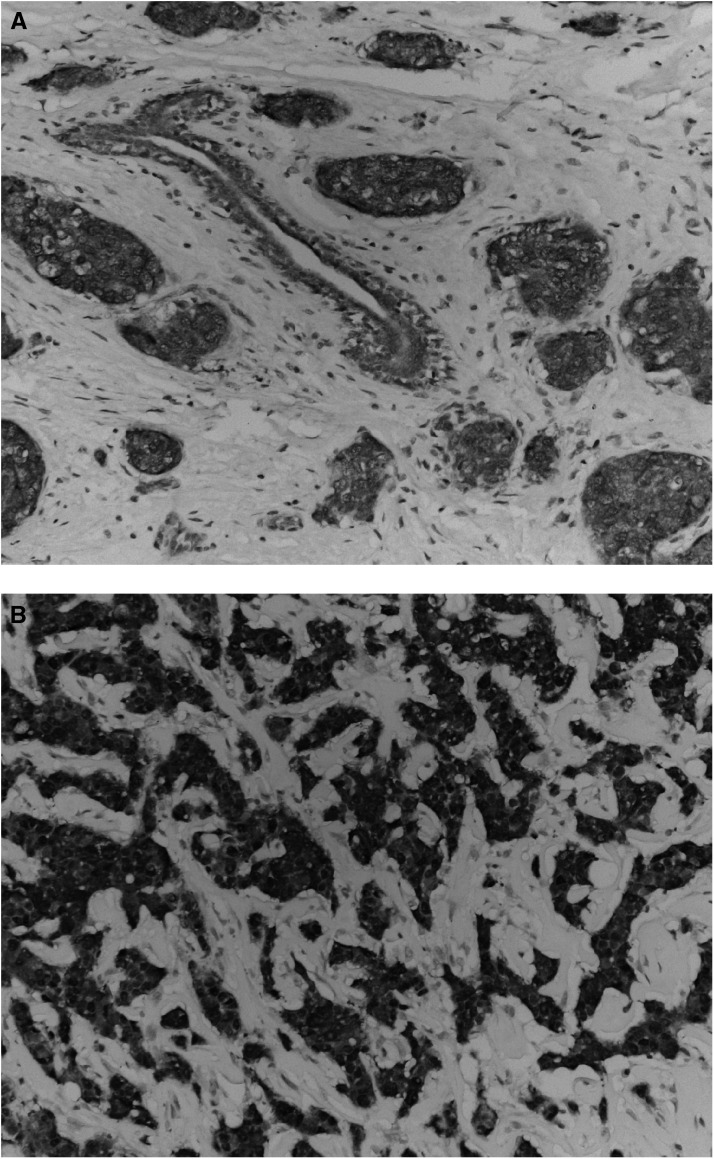
(**A** & **B**) Two cases of PS6K overexpressing breast cancer tumours.

.

### Univariate analyses

In the univariate analyses, we could not confirm a significant association between PS6K overexpression and overall survival (Table 3

**Table 3:** Univariate analyses all patients (*N*=430)

	**Locoregional control**			**Distant disease-free survival**			**Overall survival**		
**Marker**	**Hazard ratio**	**95% CI**	***P*-value**	**Hazard ratio**	**95% CI**	***P*-value**	**Hazard ratio**	**95% CI**	***P*-value**
**PS6K+**	2.50	1.30–4.81	**0.006**	1.80	1.08–3.01	**0.025**	1.27	0.61–2.64	0.526
ER+	1.38	0.50–3.79	0.536	0.54	0.33–0.90	**0.017**	0.50	0.27–0.90	**0.021**
PgR+	1.31	0.70–2.45	0.403	0.81	0.54–1.20	0.287	0.55	0.35–0.88	**0.013**
HER2+	1.64	0.89–3.01	0.111	0.84	0.56–1.60	0.95	1.17	0.63–2.17	0.617
Ki 67+	0.96	0.58–1.58	0.856	2.24	1.54–3.25	**<0.0001**	3.74	2.22–6.27	**<0.0001**
									
Grade									
I *vs* I	1.42	0.81–2.48	0.216	1.75	1.10–2.77	**0.018**	3.46	1.70–7.08	**0.0007**
II *vs* I	0.93	0.48–1.80	0.820	2.28	1.41–3.60	**0.0004**	5.09	2.53–10.26	**<0.0001**
									
Diameter									
*r*>2 cm	1.13	0.68–1.91	0.651	1.61	1.12–2.31	**0.011**	2.43	1.54–3.85	**0.0001**
p53+	1.85	1.07–3.18	**0.027**	1.07	0.69–1.67	0.763	1.47	0.87–2.46	0.148

). However, PS6K overexpression was a significant predictor for increased risk of locoregional recurrence (HR 2.50, 95% CI 1.30–4.81, *P*=0.006) and of developing distant metastases (HR 1.80, 95% CI 1.08–3.01, *P*=0.025).

### Multivariate analyses

Apart from PS6K, p53 was the only significant risk factor for locoregional recurrence in the univariate analysis. When including these two factors in a multivariate model, PS6K appears as the only independent prognostic factor for locoregional control predicting a poor control rate in PS6K overexpressing cases (HR 2.67, 95% CI 1.39–5.14, *P*=0.003, Table 4

**Table 4:** Multivariate analyses all patients (*N*=430)

**Locoregional recurrence**			
**Marker**	**Hazard ratio**	**95% CI**	***P*-value**
**PS6K+**	2.67	1.39–5.14	**0.003**
p53 positivity	1.67	0.95–2.96	0.076
			
**Marker**	**Risk ratio**	**95% CI**	***P*-value**
*Distant disease-free survival*			
**PS6K+**	1.52	0.87–2.64	0.139
ER+	0.93	0.50–2.91	0.810
Ki67 (⩾20%)	1.79	1.11–2.91	**0.018**
Grade III *vs* I/II	1.07	0.78–1.45	0.689
Diameter >2 cm	1.29	0.86–1.94	0.221
			
*Overall survival*			
ER+	1.17	0.56–2.43	0.685
PgR+	1.01	0.57–1.80	0.972
Ki67 (⩾20%)	2.84	1.44–5.59	**0.003**
Grade III *vs* I/II	1.33	0.88–2.00	0.174
Diameter >2 cm	1.63	0.99–2.69	0.055

).

Variables significantly associated with distant disease-free survival in the univariate analysis were PS6K, ER status, Ki67, grade, and tumour diameter. In a multivariate model including all these factors, Ki-67 overexpression was the only independent prognostic factor associated with poor distant disease-free survival (HR 1.79, 95% CI 1.11–2.91, *P*=0.018, Table 4). PS6K as a prognostic factor did not remain significant in the multivariate analysis. In addition, Ki-67 overexpression was an independent significant predictor for poor overall survival.

### PS6K overexpression in patients who underwent breast-conserving treatment

In all, 368 patients underwent breast-conserving therapy. Event rates are shown in Table 5

**Table 5:** Pts who underwent breast-conserving therapy

		**Number of events**		
**Patient characteristics**	***N*=368**	**Overall survival**	**Locoregional recurrence (first event)**	**Distant disease-free survival**
*Tumour diameter* (*N* (%))				
≥2 cm	249 (68)	32 (13)	38 (15)	59 (24)
>2 cm	100 (27)	27 (27)	15 (15)	36 (36)
Unknown	19 (5)	6 (32)	5 (26)	7 (37)
				
*Histologic tumour type* (*N* (%))				
Infiltrating ductal	260 (71)	48 (18)	41 (16)	73 (28)
Infiltrating lobular	22 (6)	2 (9)	7 (32)	3 (14)
Other	79 (21)	13 (16)	8 (10)	23 (29)
Unknown	7 (2)	2 (29)	2 (29)	3 (43)
				
*Histologic tumour grade* (*N* (%))				
I	124 (34)	6 (2)	19 (15)	21 (17)
II	121 (33)	26 (21)	25 (21)	40 (33)
III	107 (29)	30 (28)	11 (10)	37 (35)
Unknown	16 (4)	3 (19)	3 (19)	4 (25)
				
*ER status* (*N* (%))				
Positive	319 (87)	52 (16)	51 (16)	85 (27)
Negative	38 (10)	10 (26)	4 (11)	13 (34)
Unknown	11 (3)	3 (27)	3 (27)	4 (37)
				
*PgR status* (*N* (%))				
Positive	268 (73)	45 (17)	45 (17)	77 (29)
Negative	87 (24)	18 (21)	11 (13)	22 (25)
Unknown	13 (4)	2 (15)	2 (15)	3 (23)
				
*HER2 overexpression* (*N* (%))				
Negative	312 (85)	54 (17)	44 (14)	87 (28)
Positive	48 (13)	9 (19)	12 (25)	12 (25)
Unknown	8 (2)	2 (25)	2 (25)	3 (38)
				
*p53 expression* (*N* (%))				
Negative	290 (79)	47 (16)	42 (14)	77 (27)
Positive	70 (19)	16 (23)	14 (20)	22 (31)
Unknown	8 (2)	2 (25)	2 (25)	3 (38)
				
*Ki-67* (*N* (%))				
Negative	175 (48)	16 (9)	32 (18)	33 (19)
Positive	181 (49)	47 (26)	23 (13)	65 (36)
Unknown	12 (3)	2 (17)	3 (25)	4 (33)
				
*PS6K*				
Negative	322 (88)	54 (17)	45 (14)	83 (26)
Positive	30 (8)	7 (23)	10 (33)	13 (43)
Unknown	16 (4)	4 (25)	3 (19)	6 (38)

. The prognostic impact of PS6K was similar to that of the overall population. PS6K remained a predictor of poor locoregional control (HR 2.83, 95% CI 1.42–5.62, *P*=0.003) but not for overall survival (HR1.44, 95 CI 0.66–3.18, *P*=0.36) (Table 6

**Table 6:** Univariate analyses (Pts who received breast-conserving therapy *n*=368)

	**Locoregional control**			**Distant disease-free survival**			**Overall survival**		
**Marker**	**Hazard ratio**	**95% CI**	***P*-value**	**Hazard ratio**	**95% CI**	***P*-value**	**Hazard ratio**	**95% CI**	***P*-value**
PS6K+	2.83	1.42–5.62	**0.003**	1.80	1.00–3.23	**0.049**	1.44	0.66–3.18	0.360
ER+	1.22	0.44–3.38	0.701	0.65	0.36–1.16	0.146	0.54	0.27–1.06	0.071
PgR+	1.28	0.66–2.48	0.461	1.09	0.68–1.75	0.728	0.77	0.45–133	0.346
HER2+	1.79	0.94–3.39	0.075	0.86	0.47–1.57	0.617	1.07	0.53–2.16	0.862
Ki 67+	0.86	0.50–1.47	0.582	2.31	1.52–3.52	**<0.0001**	3.40	1.93–6.02	**<0.0001**
*Grade*									
I *vs* I	1.58	0.87–2.86	0.136	2.18	1.29–3.70	**0.004**	4.94	2.03–12.01	**0.0004**
II *vs* II	0.86	0.41–1.80	0.683	2.52	1.47–4.30	**0.0007**	7.12	2.96–17.11	**<0.0001**
									
*Diameter*									
*R*>2 cm	1.14	0.63–2.07	0.670	1.72	1.14–2.60	**0.011**	2.24	1.34–3.75	**0.002**
p53+	1.47	0.81–2.70	0.209	1.20	0.74–1.92	0.460	1.44	0.82–2.54	0.209

). In the multivariate analyses, Ki67 remained an independent predictor for distant disease (RR 1.78, 95% CI 1.03–3.07, *P*=0.038). Tumour grade remained an independent prognostic factor for poor survival (RR 1.63, 95% CI 1.04–2.53, *P*=0.032) (Table 7

**Table 7:** Multivariate analyses (Pts who received breast conserving therapy *n*=368)

**Marker**	**Risk ratio**	**95% CI**	***P*-value**
*Locoregional recurrence*			
**PS6K+**	2.83	1.42–5.62	**0.003**
			
*Distant disease-free survival*			
**PS6K+**	1.54	0.83–2.88	0.174
Ki67 (⩾20%)	1.78	1.03–3.07	**0.038**
Grade III *vs* I/II	1.17	0.83–1.64	0.369
Diameter >2 cm	1.24	0.78–1.98	0.365
			
*Overall survival*			
Ki67+	2.01	0.96–4.22	0.066
Grade III *vs* I/II	1.63	1.04–2.53	**0.032**
Diameter >2 cm	1.44	0.83–2.48	0.194

).

### FISH

A tissue microarray (TMA) was constructed from 12 tumours that showed PS6K overexpression, as assessed by immunohistochemistry. Amplification was studied using FISH by hybridising the TMA to a PS6K BAC probe and a CEP17 chromosome 17 centromeric probe. Probe signals and CEP17 signals were counted in each nucleus and a ratio of mean probe signal to mean CEP17 signal was calculated. Ratios of ⩾2 were scored as amplification. Eight of the 12 tumours with PS6K overexpression (75%) showed PS6K gene amplification, which is in accordance with the data shown by Barlund *et al* (2000a).

### Correlation between HER2 and PS6K

As the PS6K gene and the HER2 gene are both located on chromosome 17, and amplification has been reported to occur in both genes simultaneously, we studied the correlation of PS6K expression and HER2 expression and between PS6K expression and Ki67 expression, respectively. Based on available data, we found a significant association between PS6K and HER2 expression (Fisher's exact test (two sided) *P*=0.01), whereas no significant association was found between PS6K positivity and Ki67 positivity (Fisher's exact test (two sided) *P*=0.24).

## DISCUSSION

We have found that P70 S6 kinase protein overexpression in breast cancer is associated with increased risk of locoregional recurrence. To our knowledge, no other oncogenic markers as predictors of locoregional recurrence have been identified previously. At present, the common risk factors for local control after breast-conserving treatment are: patient age, margin status, and the presence of an extensive intraductal component (De la Rochefordiere *et al*, 1993; Elkhuizen *et al*, 1998; Voogd *et al*, 1999, 2001). The addition of new predictive markers for locoregional recurrence may help in guiding the optimal type of local therapy. This is of particular importance since local therapy does not only have an impact on locoregional control but also on survival (Shukla *et al*, 1999; Early Breast Cancer Trialists' Collaborative Group (EBCTCG), 2000).

P70 S6 kinase protein overexpression was associated with an increased risk of locoregional recurrence when all patients were analysed. The majority of the patients (*N*=368) underwent breast-conserving treatment. When these patients were analysed separately. PS6K remained an independent predictor of locoregional recurrence. In the univariate analysis, p53 overexpression was also associated with an increased risk of locoregional recurrence (HR 1.85, *P*=0.027); however, this was not the case for the subset of patients who underwent breast-conserving therapy (data not shown). In addition, 5-year follow-up results concerning the impact of P53 and PS6K status on locoregional control demonstrate similar results. Locoregional control rates at 5 years of follow-up are 93% (95% CI 92.3–94.2) in P53 negative *vs*. 84% (95% CI 81.7–87.2) in P53-positive patients and 93% (95% CI 91.8–93.7) *vs* 83.3% (95% CI 79.0–87.5), respectively.

Several studies have examined the relation between P53 overexpression and local breast tumour recurrence. A case–control study of 66 women with local breast tumour relapse following lumpectomy and radiation therapy showed that p53 overexpression was an independent predictive factor for ipsilateral breast tumour recurrence (IBTR) (Noguchi *et al*, 1997). Recent studies conducted by Turner *et al* (2000) and Zellars *et al* (2000) demonstrated predictive significance of P53 overexpression for locoregional recurrence in patients who underwent breast-conserving therapy, as well as in patients who underwent mastectomy. Turner and colleagues showed in a matched case-control study comprising 47 cases and 47 controls that overexpression of P53 had prognostic significance in respect to IBTR following lumpectomy and radiotherapy (*P*=0.003). Zellars and co-workers demonstrated in 1530 mastectomy-treated breast cancer patients of whom 259 received adjuvant radiotherapy that P53 overexpression was independently associated with a significantly increased local failure rate in patients treated with mastectomy, with (RR 2.5, 95% CI 1.1–5.7) or without (RR 1.7, 95% CI 1.2–2.4) radiotherapy. Although, in our series, P53 lost its prognostic significance in the multivariate analysis, a trend still remained, suggesting worse locoregional recurrence rates in P53-overexpressing tumours (RR 1.67, 95% CI 0.95–2.96).

Barlund *et al* (2000a) analysed RPS6KB1 amplification using FISH in 668 informative primary breast tumours. In all, 9% of the tumours showed amplification of the RPS6KB1 gene. In their series, PS6K was significantly associated with poor survival (*P*=0.0021). In addition, the authors analysed overexpression in a subset of 445 primary breast tumours. P70 S6 kinase protein staining of cytoplasm was subjectively scored into four groups: negative (no staining), weak, moderate, or strong staining. For statistical analyses, the data were combined into two groups: low expression (negative or weak staining) and high expression (moderate or strong staining). High expression was seen in 15.6%. There was a statistically significant association between RPS6KB1 amplification and high P70 S6 kinase protein expression (*P*=0.0004), with 41% of the amplified tumours (FISH) exhibiting high PS6K expression, and overexpression of PS6K was associated with poor survival (*P*=0.0083) as well. Our results suggest an even stronger association between amplification and expression, albeit with not enough data to make a sound statistical comparison. Moreover, the authors found that patients showing both PS6K and HER2 amplification had a significant worse prognosis in terms of breast cancer-specific survival than those with no amplification or amplification of only one of the genes.

These results together with our data suggest that P70 S6 kinase protein overexpression may be an important predictor of not only worse survival but also of poor locoregional control.
